# Processing of single-photon responses in the mammalian On and Off retinal pathways at the sensitivity limit of vision

**DOI:** 10.1098/rstb.2016.0073

**Published:** 2017-04-05

**Authors:** Daisuke Takeshita, Lina Smeds, Petri Ala-Laurila

**Affiliations:** 1 Department of Biosciences, University of Helsinki, PO Box 65, 00014 University of Helsinki, Finland; 2 Department of Neuroscience and Biomedical Engineering, Aalto University School of Science, PO Box 12200, 00076 Aalto, Finland

**Keywords:** visual sensitivity, On and Off retinal ganglion cells, scotopic vision, visual threshold, physical limits, linear and nonlinear signal processing

## Abstract

Visually guided behaviour at its sensitivity limit relies on single-photon responses originating in a small number of rod photoreceptors. For decades, researchers have debated the neural mechanisms and noise sources that underlie this striking sensitivity. To address this question, we need to understand the constraints arising from the retinal output signals provided by distinct retinal ganglion cell types. It has recently been shown in the primate retina that On and Off parasol ganglion cells, the cell types likely to underlie light detection at the absolute visual threshold, differ fundamentally not only in response polarity, but also in the way they handle single-photon responses originating in rods. The On pathway provides the brain with a thresholded, low-noise readout and the Off pathway with a noisy, linear readout. We outline the mechanistic basis of these different coding strategies and analyse their implications for detecting the weakest light signals. We show that high-fidelity, nonlinear signal processing in the On pathway comes with costs: more single-photon responses are lost and their propagation is delayed compared with the Off pathway. On the other hand, the responses of On ganglion cells allow better intensity discrimination compared with the Off ganglion cell responses near visual threshold.

This article is part of the themed issue ‘Vision in dim light’.

## 1. Introduction

Vision at its sensitivity limit relies on a small number of photons absorbed among hundreds of rod photoreceptors. These sparse signals originating in rods are transmitted through the mammalian retina via a well-defined neural circuitry. The quantal nature of light and the randomness of photon arrivals set fundamental constraints on the detectability of the weakest light signals [1] (for review, see [2]). Another constraint is set by noise generated by the rods themselves. The behavioural performance of dark-adapted humans and many other species gets remarkably close to the limits posed by the statistics of discrete photon absorptions and rod noise [3–6].

Over the last decades, a great deal has been learned about the processing of single-photon responses (SPRs) in the vertebrate retina. More than 70 years ago, classic human psychophysics experiments indicated that a small number of absorbed photons was enough to cause a behaviourally measurable response. Because the photons were spatially distributed over several hundreds of rods in these experiments, the results also indicated that single rods must be able to encode single photons [5]. About 40 years later, SPRs were isolated physiologically in toad rods by the suction pipette technique [7]. Since then, the mechanistic basis of the reproducibility of SPRs has been chara­cterized in great detail [8–12]. Great progress has also been made in characterizing the primary noise sources of rod photoreceptors consisting of spontaneous activations of visual pigments and downstream phototransduction noise [13–15]. More recently, work on retinal circuits has unravelled many ­­of the key mechanisms that minimize the impact of retinal noise and increase the reliability of SPR processing in mammalian retinal circuits [16–20]. Similarly, classical work on the retinal output neurons, the retinal ganglion cells (RGCs), has provided insights about the sensitivity limits as well as the response properties of these cells [21–23]. Finally, by now, the behavioural performance of several vertebrate species, including amphibians and mice has been characterized at visual threshold [6,24,25].

Despite these great advances, one important issue has not been addressed to any large extent in the previous literature. There are two fundamentally different retinal outputs provided by On and Off type RGCs that set different constraints on the detectability of the weakest light signals. On RGCs respond to light by increasing their firing rate, whereas Off RGCs decrease their firing rate in response to light increments. On and Off retinal pathways also differ fundamentally in the way they handle SPRs originating in rods. At least in primates, the On pathway has a nonlinear coincidence detection mechanism for SPRs in the inner retina, whereas the Off pathway does not [16]. Thus, the brain receives two different readouts of the SPRs originating in rods: a thresholded, low-noise readout via the On pathway and a noisy, linear readout via the Off pathway. Most models that have been used for estimating the minimum number of photons needed for detection assume that retinal signal processing at the absolute threshold is essentially linear (for review, see [26]). This is consistent with the current knowledge of the Off pathway but not the On pathway. Currently, we do not know how behaviour depends on these two different output signals at visual threshold.

Here, we outline the mechanistic basis of the different coding strategies in the On and Off pathways and analyse their implications for downstream circuits in detecting the weakest light signals. We focus on three functional aspects: the speed of encoding SPRs originating in rods, the sensitivity limit for detecting light and the sensitivity for discriminating light increments of different intensities near visual threshold. We show that the high-fidelity signal processing in the On pathway comes with two distinct costs: first, a fraction of transmitted SPRs is lost in the inner-retinal nonlinearity of the On pathway, causing a decrease in sensitivity. Second, the nonlinearity causes a delay in signal propagation via the On pathway compared with the Off pathway. On the other hand, the responses of On RGCs provide better discrimination between different light intensities near visual threshold than do those of Off RGCs.

## 2. Asymmetric signal propagation through the mammalian retina via the On and Off pathways

**Figure 1.**
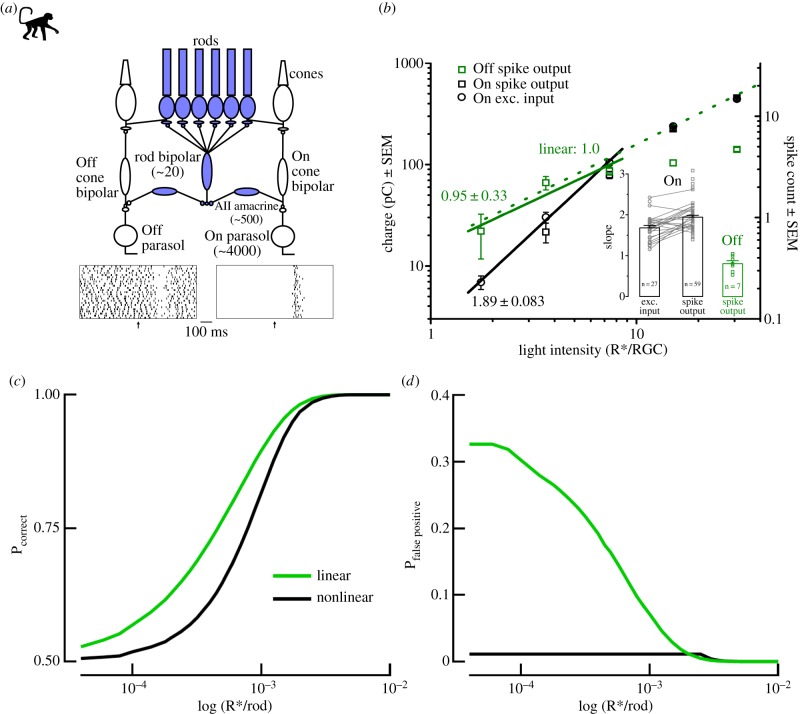
Nonlinearity in the On but not in the Off pathway near the absolute threshold. (*a*) Top: schematics of the rod bipolar pathway in the primate retina. Near the absolute threshold, the primary Off and On pathways (rod bipolar pathway) share the circuitry up to the AII amacrine cell (highlighted in blue). The synapse between On cone bipolar cells and ganglion cells not only operates as a thresholding nonlinearity to reduce noise, but also limits information about single photons. The numbers shown in the diagram indicate the number of rods converging on a particular cell type. Spike responses to dim flashes are shown for an Off (left) and On parasol cell (right) at the bottom. (*b*) The stimulus–response relationship for primate Off and On parasol cells near absolute threshold. At very low light levels (a few R* per RGC), both the spiking responses (black squares) and excitatory input currents (black circles) of On cells show supralinearity, while the spiking responses of Off cells (green squares) show a linear relationship. The dashed line shows a linear relationship as a reference. Inset: the slopes of the stimulus-response relationship measured at low-light intensities for the excitatory synaptic current to On (left), spike response of On (middle) and spike response of Off (right) parasol cells. (*c*) Dim-flash detection performance predicted by a nonlinear (black) and linear (green) model. The nonlinear model is in line with the On parasol responses while the linear model predicts Off cell responses. (*d*) The false-positive rates predicted by the model in (*c*). Adapted with permission from Ala-Laurila & Rieke [16].

The outputs provided by different RGC types can be divided into two major classes: On and Off type RGCs [27]. At cone-driven (photopic) light levels, signals in the vertebrate retina diverge into parallel On and Off pathways already at the first synapse. On bipolar cells depolarize in response to light increments, whereas Off bipolar cells hyperpolarize [28]. The contributions of 14 different bipolar cell types [29] driving RGC signals can vary depending on the background light levels: some RGC types can turn from On to Off or vice versa as the light levels increase owing to changes in the relative contributions of On and Off type bipolar cells [30,31]. At mesopic, and even at rod-driven (scotopic) light levels higher than visual threshold, multiple pathways mediating rod and/or cone signals through the retina can be active at the same time [32]. However, near the dark-adapted visual threshold, rod-driven signals propagate through the mammalian retina via the so-called rod bipolar pathway [33–36] (figure 1*a*): rod → rod bipolar cell → AII amacrine cell → On and/or Off cone bipolar cell → RGC. In these conditions, On and Off RGCs share the same pathway up to the AII amacrine cells, so that signals from rod SPRs diverge into depolarizing (On) and hyperpolarizing (Off) responses only at the AII output in the inner retina. This is the case of interest here: what are the functional consequences of the On and Off pathway asymmetries at the lowest light levels, where sparse rod-driven signals traverse the mammalian retina via the rod bipolar pathway?

Several studies have shown that the On and Off pathways of the mammalian retina are not simply mirror images of each other with opposite polarities in response to negative and positive contrast in visual scenes. In photopic conditions, various asymmetries have been found in their spatio-temporal response properties indicating that they carry different information on visual scenes. These include receptive field size [37,38], response kinetics [37] and the degree of the nonlinearity of their inhibitory and excitatory inputs [37,39]. Asymmetries between On and Off pathways have also been found in scotopic conditions, where (mouse) Off RGCs propagate information at higher frequencies and with faster kinetics than On cells [40]. However, the background light level used in that study (approx. 0.3 photoisomerizations per rod per second, R* per rod per second) was still approximately 30 times higher than the level corresponding to the spontaneous activation rate of rhodopsin in mouse rods [41] and thus far from representative of the conditions comprising detection of the weakest light increments in the dark.

In those conditions, close to the absolute visual threshold, another mechanism creates a crucial functional difference between the two pathways, as recently described by Ala-Laurila & Rieke [16]. The On pathway shows highly nonlinear response properties, whereas the Off pathway is essentially linear (figure 1*a*,*b*). The mechanism underlying this asymmetry resides in the last synapse of the On pathway, located between On cone bipolar cells and On RGCs, and operates as a coincidence detector that passes signals only when two or more SPRs occur simultaneously in the receptive field of an On cone bipolar cell consisting of approximately 1000 rods [16].

This nonlinearity causes a significant asymmetry between the On and Off retinal pathways. It eliminates a significant fraction of SPRs in the On pathway, causing a decrease in sensitivity for the weakest light signals. Its impact on retinal output is demonstrated by a model constructed in Ala-Laurila & Rieke [16]. Figure 1*c*,*d* illustrates this model. The black lines show model performance for nonlinear signal processing (On RGCs), and the green lines for linear processing (Off RGCs) in a two-alternative forced-choice task of discriminating weak light pulses from neural noise. As shown in figure 1*c*, the nonlinearity shifts the probability of correct choices to the right, so that for example the flash strength needed for 75% correct choices increases by approximately 40%. For the weakest light intensities, the sensitivity decrease is even larger. However, the false-positive rate (i.e. responses when there is no flash) decreases by more than 30-fold (figure 1*d*). The nonlinearity effectively eliminates neural noise, so that the On pathway provides the brain with an essentially noiseless estimate of the weakest light signals at the cost of losing most SPRs, whereas the Off pathway provides a linear and noisier output with higher sensitivity. Thus, the two pathways make different trade-offs between sensitivity and reliability.

## 3. The most sensitive retinal ganglion cells and their response properties at visual threshold

Currently, more than 30 distinct RGC types have been identified in the mouse retina [42] and approximately 20 types in the primate retina [43] (for review, see [44]). Although the absolute sensitivity limits of all distinct RGC types have not been measured systematically, alpha retinal ganglion cells (αRGCs) in the mouse retina and parasol ganglion cells in the primate retina are currently the most promising candidates for correlating absolute sensitivities of the retinal output and behaviour. They receive abundant rod input [45,46] and provide information on subtle changes in contrast [47–50]. In the mouse, αRGCs have the highest sensitivity among all RGC types tested so far at scotopic light levels (cell types defined by clustering based on their responses to spatial stimuli as well as morphology; Dr Greg Schwartz 2016, personal communication).

Alpha RGCs were originally described in studies of the cat retina and morphologically described as RGCs with large somas and radiating dendritic arbours [51,52]. Although αRGCs have been identified across many species, there are some differences between species. In the primate retina, On and Off parasol cells, originally described by Polyak [53] (for review, see [54]), are the closest homologues of the cat αRGCs [55–57]. Similarly as in the cat retina, only one paramorphic pair (On and Off parasols) has been found in the primate retina. In the mouse retina, four types of αRGCs are presently distinguished. Three of them belong to previously described classes (On- and Off-sustained and Off-transient) [58]. A fourth type called On-transient αRGC has recently been added [59]. Mouse On-sustained αRGCs, but not Off αRGCs, have also been found to express melanopsin endowing them with intrinsic photosensitivity [60,61]. Although melanopsin generates robust responses to single absorbed photons, its density is so low that it cannot contribute to light detection at the sensitivity limit of αRGCs, where rod-driven signals dominate owing to a much higher photon capture rate in rods [62,63]. In this paper, we use the term On and Off RGCs exclusively to refer to On and Off parasols in the primate retina and On- and Off-sustained αRGCs in the mouse.

It should be noted that older literature on mammalian RGC performance at the absolute visual threshold applies various classification schemes mostly for historical and technical reasons. The early work was carried out on RGCs in the anaesthetized cat [21,64,65] (for review, see [26]). Cell identification was then not accompanied by morphological classification, as cells were classified as Y and X based on their response properties: Y cells show nonlinear spatial summation at cone-driven light levels, whereas X cells show linear spatial summation [66]. The Y cells have response properties which are mostly consistent with what is now known of αRGCs and parasol RGCs [67–70].

### (a) Response properties of On and Off retinal ganglion cells

In the dark, On and Off RGCs have fundamentally different spontaneous activity and light-evoked response properties. This is demonstrated in the raster plots in figure 1*a* which show the responses of primate On and Off parasols to a flash eliciting on average approximately seven photoisomerizations in the ganglion cell receptive field (R* per RGC). On parasol cells in the primate retina are almost silent in the dark (less than 0.5 Hz firing rate), whereas Off parasols have a substantial intrinsic firing rate (approx. 20 Hz) [16]. Mouse αRGCs show a similar trend in their spontaneous activity: On-sustained αRGCs have very low intrinsic firing rates, whereas both Off-sustained and Off-transient αRGCs show robust spontaneous spiking activity [58,71]. Off-transient cells have on average slightly lower firing rates in the dark compared with Off-sustained cells in wild-type (C57BL/6) mice: 22 versus 38 Hz [71]. The spontaneous firing rates for the αRGCs (CBA mouse line expressing melatonin in the retina) of On and Off αRGCs in the dark are 0.05 ± 0.09 (On-sustained αRGCs, *n* = 16) and 84 ± 11 Hz (Off-sustained αRGCs, *n* = 29) [72]. The very low spontaneous firing rates of On cells are consistent with the thresholding nonlinearity in the inner retina discussed above, blocking most signals from spontaneous activation of rhodopsin molecules in rods. Indeed, a dim background light causing on average only one activated rod among approximately 1000 rods in the integration time of the inner-retinal nonlinearity can relieve this nonlinearity in the On parasol cells, leading to significant maintained firing rates [16]. It should be noted, however, that the earlier measurements in the cat retina are not in line with the results obtained in the *in vitro* flat-mounted mouse and primate retinas. In retinas of anaesthetized cats, both On and Off αRGCs show significantly higher spontaneous spiking activity in the dark [64]. There are several possible explanations for this difference: (i) different recording conditions (*in vitro* retina versus eye *in situ*), (ii) the anaesthetics used in the cat recordings and (iii) a species difference. Resolving this question requires future investigation (see Future perspectives).

In addition to the differences in spontaneous firing rates, On and Off αRGCs differ fundamentally in their responses to the SPRs originating in rods. Results from the primate retina show that Off RGCs with high intrinsic firing rates respond by gaps in their tonic firing that scale linearly with the number of rhodopsin activations in their receptive fields (R* per RGC). On RGCs with almost no intrinsic firing rate respond to light increments by increasing their firing rate and integrate SPRs nonlinearly (figure 1*b*). These are the primary differences in the response properties. In addition, there are some notable differences between mouse Off-sustained and Off-transient αRGCs. The latter have a transient increase in firing rate right after the gap caused by a light stimulus [34,71]. The mechanism underlying the difference between sustained and transient Off cell response properties is not fully resolved. Current evidence points towards an undefined amacrine cell input via gap junctions contributing to Off-transient cell responses at low-light levels [71].

### (b) Absolute threshold of On and Off retinal ganglion cells

Both On and Off RGCs carry rich information about the weakest light signals. Off cells are somewhat more sensitive than On cells but have a higher error rate in their gap-based coding. The absolute threshold for primate On and Off parasol cells in a two-alternative forced-choice task is extremely close to the limits posed by the quantal nature of light: On parasols reach 75% correct choices at a light level corresponding to approximately 0.0008 R* per rod per flash (mean, *n* = 6) and Off parasols at a light level corresponding to approximately 0.0004 R* per rod per flash (mean, *n* = 5). Assuming 4000 rods in the receptive fields of On and Off parasols in the dark [73], these light levels correspond to approximately 3 and 2 R* in the entire receptive field of On and Off RGCs, respectively (data from [16]). For mouse On and Off αRGCs (CBA mouse line, [72]) the absolute threshold is approximately 1 log unit higher than for the On and Off parasols in the primate retina: approximately 0.006 R* per rod per flash (mean, *n* = 26) for On-sustained αRGCs, and approximately 0.003 R* per rod per flash for Off-sustained αRGCs (mean, *n* = 46). Assuming approximately 10 000-fold rod convergence for mouse αRGCs in the dark, these thresholds correspond to approximately 60 and 30 R* in the entire receptive fields of On and Off αRGCs, respectively. Murphy & Rieke [71] report fairly similar values for the Off-sustained αRGCs and Off-transient αRGCs in C57BL/6 mice: approximately 0.002 R* per rod per flash (Off-sustained αRGCs) and approximately 0.001 R* per rod per flash (Off-transient αRGCs).

The key question is how behaviour relates to the two fundamentally different codes presented by On and Off αRGCs at visual threshold. Linking behavioural performance to the On and Off retinal outputs has not been done before. Doing this at the absolute visual threshold appears as a future task of fundamental importance, but it will be experimentally highly challenging, as the sensitivities of the two pathways are so similar. However, their distinct coding properties will constrain behaviour and downstream computations in different ways. In the following, we analyse the constraints posed by these two different coding mechanisms, focusing on the timing of detecting the weakest light pulses and the discriminability of different light intensities in the primate and mouse retina. We will then discuss the implications of On and Off pathway mechanisms on correlated activity and noise in retinal outputs at the lowest light levels and the implications for behavioural performance at visual threshold.

## 4. Trade-off between response reliability and speed: difference between the On and Off pathways

Thresholding nonlinearities, like many neural mechanisms that improve signal reliability, come with trade-offs. First, there is a trade-off between retaining SPRs and eliminating noise by thresholding in the rod bipolar pathway. Field & Rieke estimated that even approximately 50% of the SPRs originating in rods are lost owing to the thresholding nonlinearity in the rod to rod bipolar cell synapse in the mouse retina and somewhat less in the primate retina [17,26]. Ala-Laurila & Rieke showed that the thresholding nonlinearity in the inner retina of the On pathway eliminates up to 90% of all SPRs and/or spontaneous ‘dark’ events arriving at this circuit location from the pool of approximately 1000 rods [16]. Yet, most signals arising from *coincident* arrivals of multiple SPRs are allowed to pass while most of the neural noise is eliminated. Second, thresholding nonlinearities must also set a constraint on the speed of mediating information about SPRs. This latter trade-off has not been discussed previously at the absolute sensitivity limit of On and Off pathways.

*How much is detection of the weakest signals slowed down by the nonlinearity in the On pathway?* We address this question by studying the impact of the nonlinear signal processing on the delay in encoding SPRs at the inputs of On parasol ganglion cells in the primate retina. In addition, we compare mouse On and Off αRGC spike responses in terms of the speed of mediating information about the weakest light stimuli.

**Figure 2.**
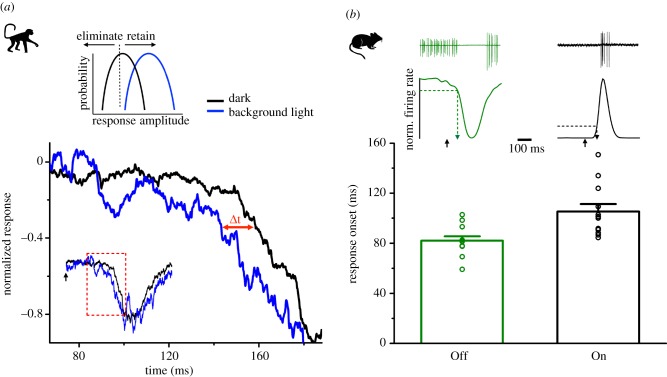
Response delays caused by thresholding in the On pathway. (*a*) Dim background light removes the effect of thresholding in a primate On parasol cell. Top: a simple model illustrating how dim background light relieves the thresholding nonlinearity. In the dark, some of the light responses are eliminated by thresholding (black). Dim background light shifts the whole response distribution above the threshold (blue). Bottom: excitatory synaptic current elicited by a brief flash in darkness (black) and on a dim background (blue). The flash intensity is 2 R* per RGC, assuming the convergence of 4000 rods per RGC. The dim background pushes the baseline above the threshold and the onset of the signal moves earlier (Δt). (*b*) Mouse On-sustained αRGCs respond more slowly to dim light flashes than Off-sustained αRGCs. Top: an example of average dim light spike response and the response peri-stimulus time histogram of an Off (green; flash intensity 0.0085 R* per rod per flash) and On cell (black; 0.0083 R* per rod per flash). The black arrow at the bottom indicates the stimulus onset. The response onset for a given light intensity, indicated with the dashed arrow, is defined as the timing when the normalized instantaneous firing rate deviated from the baseline by 20% of the entire response. Bottom: the response onset of mouse Off and On RGCs determined at 0.0087 R* per rod per flash. Bar graphs and circles show mean ± s.e.m. and individual values, respectively. Response onset for mouse Off cells was 82.1 ± 3.5 ms (*n* = 12) and for On cells 105.3 ± 6.0 ms (*n* = 12). The two onset values differ significantly (*p* < 0.003, rank-sum test).

The top graph in figure 2*a* demonstrates the basic principle of a thresholding nonlinearity: a threshold based on a criterion amplitude is used to segregate signals from noise. The black trace illustrates an amplitude distribution of weak signals in the dark. A background light that shifts the response distribution above the threshold (blue line) will relieve such a nonlinearity. We analysed whether the information about the weakest light signals is available faster in the presence of a background light relieving the inner-retinal nonlinearity. The analysis refers to the excitatory input of a primate On parasol RGC. Given that coincidence of two or more photons at the inner-retinal nonlinearity is needed for the signal to pass [16], the prediction is that there will be a delay owing to the time it takes for the first SPR to reach its peak and allow the coincident SPRs to pass the threshold.

The black line in the main panel of figure 2*a* shows normalized excitatory input currents of an On parasol RGC in the dark in response to a dim flash causing on average two photoisomerizations in the entire RGC receptive field. We compared the kinetics of the normalized response in the dark with a response in the presence of a background light (blue line) with an intensity (0.008 R* per rod per second) that will relieve the nonlinearity in the input currents. This background light is so dim that it cannot cause adaptation at any circuit site prior to the inner-retinal nonlinearity (see [16]). As seen, the response kinetics are indeed faster in the presence of a background light. In this example, the response reaches 30–70% of its peak value approximately 16 ms earlier than in the dark. It should be noted that the flash is given at the time 0. Thus, there is a long delay (greater than 100 ms) before any response is elicited even when the inner-retinal nonlinearity is released. This delay is the sum of all transmission delays in the rod bipolar pathway in the dark, from photon absorption up to the arrival of the signal at the RGC. Even so, the data clearly suggest that the additional delay caused by the inner-retinal nonlinearity in the On pathway is not insignificant. However, these preliminary estimates are based on a limited number of cells and will need to be supplemented by larger population data for more exact numerical values.

As a corollary, we hypothesize that the On pathway is slower in encoding dim light flashes compared with the Off pathway, which does not include any corresponding nonlinearity. Earlier work has shown that, at low scotopic light levels (approx. 2–3 R* per rod per second), changes in the excitatory synaptic input to On-sustained αRGCs and the inhibitory synaptic input to Off-transient αRGCs occur almost simultaneously [74]. However, these background light levels are a few hundred times higher than those that already override the inner-retinal nonlinearity of the primate On pathway. Here we simply wanted to compare the times it takes for the weakest light signals to reach retinal outputs via the On and Off pathways in the dark-adapted mouse retina. The top graphs of figure 2*b* show spike responses of an Off-sustained αRGC and an On-sustained αRGC to a flash eliciting on average approximately 90 photoisomerizations in the entire receptive field (assuming 10 000 rod convergence, [75]). The average normalized instantaneous firing rates are shown below the spike responses. Based on these measurements, we read the time it took to reach 20% of the maximum response amplitude for both Off and On cells (downward arrows in top panels). As shown by the population data (bottom panel) the Off cells (82 ± 3.5 ms, mean ± s.e.m., *n* = 12) were significantly faster than the On cells (105.3 ± 6.0 ms, mean ± s.e.m., *n* = 12, *p* < 0.003, rank-sum test). These data are consistent with the notion that the extra delay in encoding SPRs owing to the On pathway nonlinearity is approximately 20 ms near visual threshold. Two-alternative forced-choice analysis run over short time intervals after the flash also showed that the information about weak light flashes was available faster via the Off pathway (data not shown). Further investigations in the mouse retina will be needed to provide precise evidence of the circuit site where this delay arises. Our analysis shows that reading the gaps in firing in the Off pathway would give downstream circuits earlier access to the information of the weakest light signals. Whether such information is used in the higher brain regions remains to be seen.

## 5. Discriminability of light increments by On and Off retinal ganglion cells

Above we have outlined how On and Off αRGCs with different coding strategies carry information of the weakest light pulses close to visual threshold. As shown earlier in figure 1*c*,*d*, Off cells have slightly higher absolute sensitivity in darkness but also higher noise levels compared with On cells [16,34]. Now we ask how well spike responses of On and Off RGCs in the primate and mouse retina allow the discrimination of intensity differences of light stimuli close to the absolute threshold. How well do the gaps in firing of Off cells and the increase in the spike rates of On cells, respectively, encode *graded* information about the intensities of weak flashes?

**Figure 3.**
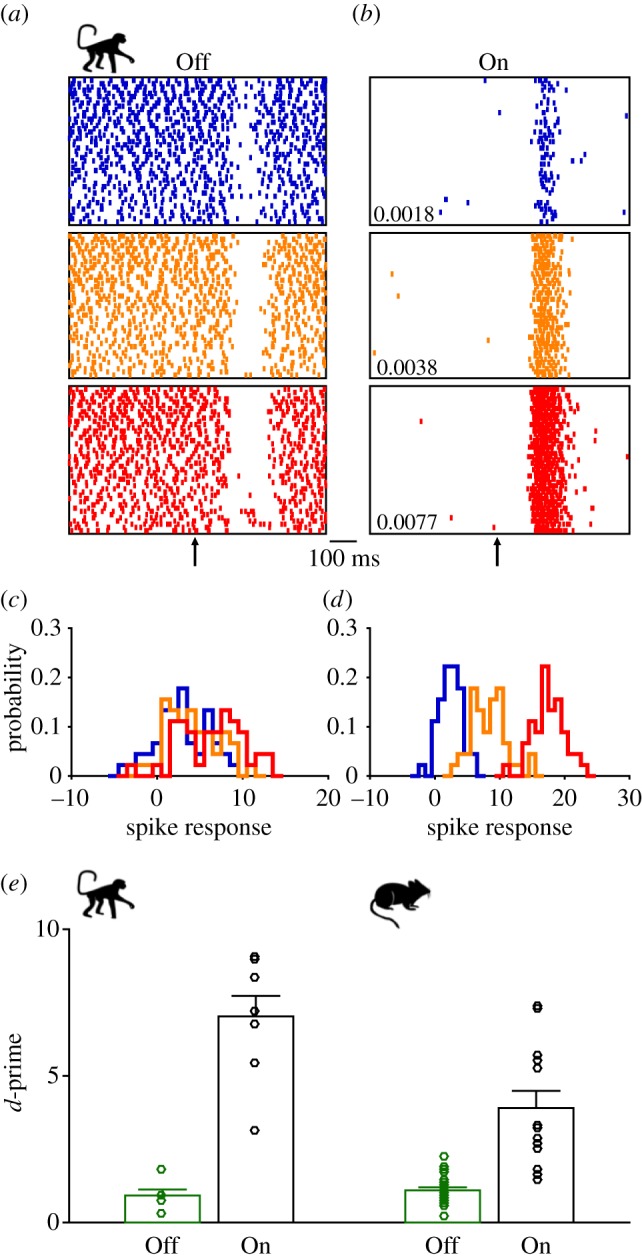
On RGCs discriminate differences in light intensity better than Off RGCs. (*a*) Primate Off parasol ganglion cell spike responses to dim flashes delivered at the time point of the arrow. Each box shows 45 trials with flashes of the nominal light intensity in R* per rod per flash indicated at the lower left corner in (*b*). (*b*) On parasol ganglion cell spike responses to the same flash intensities as (*a*). (*c*) Off parasol cell spike response distribution for the same cell and flash intensities in (*a*). The response for each trial was defined as the difference in spike count between 400 ms time windows before and after the flash. (*d*) On parasol cell spike response distribution for the same cell and flash intensities in (*b*). (*e*) The discriminability of spike responses to two different light intensities of Off (green) and On (black) parasol cells in the primate retina (left) and Off (green) and On (black) αRGCs in the mouse retina (right). The discriminability was quantified as the difference of means of two spike response distributions normalized by their standard deviations (*d*-prime). The bar graph shows mean and s.e.m. for each population. The two light intensities chosen were 0.0018 and 0.0077, and 0.0037 and 0.017 R* per rod per flash for primate parasol cells and mouse αRGCs, respectively. The *d*-prime for primate Off RGCs was 0.92 ± 0.21 (mean ± s.e.m., *n* = 6 cells), which was significantly smaller than that for primate On RGCs (7.0 ± 0.70, *n* = 8, *p* < 10^−3^, rank-sum test). The *d*-prime for mouse Off RGCs was 1.1 ± 0.1 (*n* = 24), which was significantly smaller than that for On RGCs (3.9 ± 0.58, *n* = 13, *p* < 10^−5^).

Figure 3*a*,*b* illustrates the response of Off (figure 3*a*) and On parasol RGCs (figure 3*b*) to brief flashes of three different increasing intensities (from top to bottom: 0.002, 0.004 and 0.008 R* per rod per flash) in the dark. Off parasols show spontaneous spiking in the dark and spike rate decreases in response to light flashes (figure 3*a*). On cells are almost silent in the dark and respond to flashes by spike bursts (figure 3*b*). To study the intensity dependence of Off and On cells, we defined the response as the difference in spike count in 400 ms time windows preceding and following the stimulus onset. For Off cells, the distributions of the response to the three light intensities had large overlap (figure 3*c*). By contrast, for On cells, the distributions overlapped rather little (figure 3*d*). To quantify this effect over populations, we chose a pair of stimulus intensities and measured the separation of the two response distributions using the difference of means normalized by their standard deviations, known as *d*-prime (*d’*) in signal detection theory:5.1
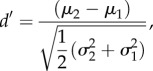
where *μ*_1_, *μ*_2_, *σ*_1_ and *σ*_2_ are the means and standard deviations of the two distributions, respectively [76]. Note that a larger *d*-prime means a larger separation in the means of two distributions with respect to their standard deviations.

This analysis was done for both primate parasol RGCs and mouse αRGCs. In both species, the *d*-prime of Off cells was significantly smaller than that of On cells (figure 3*e*), implying that discrimination of the two light intensities would be better based on the response from On cells than based on that from Off cells.

One reason why On RGCs give a better discrimination than Off RGCs is the difference in the fluctuations in the spontaneous firing rate. For On cells, the spike count in the pre-stimulus interval is almost always zero and, thus, the fluctuation is small in the dark. By contrast, in Off cells, the spike count in the pre-stimulus interval shows a Poisson-like fluctuation where the variance is equal to the mean. These fluctuations will make it more difficult for downstream circuits to discern different light intensities based on changes in spike counts from spontaneous firing. Another reason is that Off cells respond to light by decreasing their firing rate from a level maintained in darkness, which limits the dynamic range (i.e. the decrease in spike count cannot exceed the initial spiking activity). This is particularly clear in primate Off cells whose spontaneous firing rate is up to approximately 30 Hz, which is less than half of that of mouse Off-sustained alpha cells in the dark (70 Hz, CBA mouse line, [72]). As a consequence, primate Off cell responses saturate very easily for brighter stimuli. In summary, even though Off cells can detect weak flashes more sensitively than On cells, On cells discriminate different dim light intensities better than Off cells. Furthermore, as shown in figure 1*d*, On cells have a very low false-positive rate owing to the noise filtering by the thresholding nonlinearity in their excitatory input current.

## 6. Limiting noise sources, correlated activity in retinal outputs and the behavioural sensitivity limit

The asymmetry between the mammalian On and Off retinal pathways at visual threshold has several implications for the interpretation of the limiting noise sources, the noise correlations in retinal outputs, and the behaviourally measured sensitivity limit. We outline in the following how the recent finding of an inner-retinal nonlinearity in the On pathway [16] influences the current understanding of these topics.

### (a) Noise correlations and limiting noise in retinal outputs

For decades, it has been discussed what neural noise sources limit behaviourally measured visual sensitivity at the absolute threshold. Barlow [3] famously proposed that spontaneous isomerizations of visual pigments in photoreceptors cause ‘false’ signals that are indistinguishable from real photon-induced signals and thereby ultimately limit visual sensitivity. However, the evidence for reaching this limit in the mammalian retina has been suggestive rather than definitive (for review, see [26]). Comprehensive noise analysis at visual threshold has not been carried out at the level of RGC input currents. Mostly, current evidence for the pigment noise hypothesis as a limiting noise relies on RGC recordings done on anaesthetized cat retinas. High intrinsic firing rates of cat On ganglion cells have been interpreted to suggest that spontaneous pigment activations could drive the spiking activity of RGCs [21,64]. Mastronarde analysed the correlated activity between neighbouring cat RGCs in the dark and in the dim background light. He showed that the cross-correlations were consistent with a shared noise source in their receptive fields and that the frequency of these correlated events increased with increasing background light levels. The kinetics of the cross-correlations were consistent with the idea that the dominant noise could originate in spontaneous pigment activations. However, current results both in the primate and mouse ganglion cells in flat-mounted *in vitro* preparations show fundamentally different behaviour [16,18,74]. First, On RGCs are very silent in the dark, indicating that the inner-retinal nonlinearity eliminates most signals originating in spontaneous activations of visual pigments. Furthermore, the cross-correlations of both primate and mouse On RGC input currents show much faster kinetics than pigment-related events suggesting that the remaining noise in the input currents of the On ganglion cells arises from a downstream source. On the other hand, even a very weak background light can eliminate the nonlinearity in the On pathway and allow pigment noise to pass the retina to the ganglion cell outputs (P. Ala-Laurila & F. Rieke, unpublished data). All in all, the recent results suggest that very little pigment noise reaches the retinal outputs through the On pathway in the dark. On the other hand, the Off pathway, as a linear channel, could encode pigment noise via gaps in intrinsic firing rate in the dark. Thus, the mechanistic origin of the noise in the On and Off pathway in the dark is most likely different. It remains to be seen to what extent the downstream circuits and behaviour rely on one or the other pathway for detection of the weakest light pulses in darkness.

Even though noise from spontaneous pigment activations in rods does not reach the output of On RGCs, it may still play an important role in visual sensitivity, by forcing the design of retinal processing mechanisms to deal with it. This notion is supported by the location and ‘tuning’ of the inner-retinal threshold, requiring coincidence of approximately two or more events within a neural integration time of approximately 50 ms. This appears as an amazing adaptation for eliminating the pigment events occurring at rates of approximately 0.003–0.005 R* per rod per second [26], and converging from approximately 1000 rods at the site of this nonlinearity. It would be interesting to study, e.g. whether different matches of inner-retinal threshold, rod convergence and rod pigment noise might be found in other mammalian species.

### (b) Behavioural sensitivity limit

The estimates for the smallest number of R*s that humans can detect vary from a few to several tens [26,77]. Previous literature has pointed out that the models used for getting these numbers based on behavioural data are not well constrained [26], and they do not, of course, take into account the novel inner-retinal nonlinearities now known. Intriguingly, a recent study relying on a single-photon source concluded that hardly any SPRs were detected by dark-adapted humans [78] (see also [79]). These data are consistent with the idea that the detection of photons near visual threshold could rely on the On pathway where the inner-retinal nonlinearity eliminates almost all SPRs. An alternative hypothesis is that the Off pathway, passing SPRs, contributes too but that downstream nonlinearities eliminate the weakest light signals. This question could be experimentally addressed by applying background light precisely calibrated to relieve the inner-retinal nonlinearity in the On pathway and repeating the classic human frequency of seeing experiments in these conditions. Even more elegantly, one could apply the single-photon sources to test single-photon detection in the presence of such dim background light. This approach would be especially interesting in the light of a new study relying on a single-photon source and showing evidence for nonlinear interaction between individual photons right at the absolute sensitivity limit of human vision [80].

## 7. Future perspectives

In this paper, we have described how the asymmetry between the mammalian retinal On and Off pathways impacts visual processing at the lowest light levels. In the following, we outline some outstanding unsolved problems regarding vision at the absolute threshold in the mammalian retina:

— *How does behavioural performance depend on the On and Off retinal outputs near the absolute visual threshold*? Experimental approaches aiming at correlating On and Off retinal pathways to behavioural performance in the dark and at the lowest light levels in increment and decrement coding would be very valuable, but the similarity in sensitivity of the two pathways despite the very different coding strategies makes this a challenging task. Transgenic mouse models might allow us to break this similarity in sensitivities and to seek deeper understanding of the roles of On and Off pathways at visual threshold.
— *What are the absolute sensitivity limits of the distinct ganglion cell types in the mammalian retina*? It would be valuable to characterize the response properties and sensitivity limits of the currently identified RGC types (more than 30 in the mouse retina). Correlating the performance limits of various RGC types with specific visually guided behaviour tasks may lead to a deeper understanding of the role of each RGC type at low-light intensities.
— *What are the mechanistic origins of noise at low-light levels in the inputs and outputs of distinct RGC types?* Current tools allow approaching well-defined ganglion cell types. Determining the mechanistic origin of noise in the inputs and outputs of different RGC types at the lowest light levels would lead to a better understanding of the sensitivity limitations originating in the retina.
— *Where do the differences arise between the classic ganglion cell experiments on anaesthetized cats versus recent experiments on* in vitro *preparations of the mouse and primate retinas*? Seeking understanding by making recordings in identical conditions across these three model species would be very valuable to gain deeper understanding of the datasets that give so different predictions of firing rates and potential limiting noise sources in retinal outputs *in vivo*. It will be important to test the effect of the anaesthetics used in the classic cat recordings on flat-mounted *in vitro* retinal preparations.

## Data accessibility

Data is available at http://dx.doi.org/10.6084/m9.figshare.4232717.
